# Supplementary material for: Detecting and quantifying overparametrization in RNA language models with REDIAL
Source: bioRxiv. 2026 May 12:2026.05.11.724344. Preprint. [Version 1] doi: 10.64898/2026.05.11.724344 (PMC13192764; doi:10.64898/2026.05.11.724344)
Supplement: 1 [file NIHPP2026.05.11.724344V1-supplement-1.pdf]

bioRxiv preprint doi: https://doi.org/10.64898/2026.05.11.724344; this version posted May 12, 2026. The copyright holder for this preprint (which was not certified by peer review) is the author/funder, who has granted bioRxiv a license to display the preprint in perpetuity. It is made available under a CC-BY 4.0 International license.

Supplementary Information

A Comparison between Categorical Jacobian and Embedding Perturbation

"Categorical Jacobian" (CJ) algorithm is an unsupervised method proposed by Zhang *et al.*[20] to extract coevolutionary coupling from pLMs. We adapted it to work for the RNA alphabets. The largest difference between REDIAL and CJ algorithm is that CJ looks at changes in logits, and REDIAL looks at changes in the embedding (Figure 5). The details are described as follow:

Decoders for RNA language models typically produce a logit with the dimension $L \times A$, where the model has a vocabulary $\mathcal{A}$ of $A = 4$ standard tokens representing the nucleotides A, U, C, and G. Similarly, because there are a total of $L \times A$ possible single-point mutations in the sequence, a four-dimensional "Jacobian"-style tensor $\mathcal{J} \in \mathbb{R}^{L\times A\times L\times A}$ can be defined to capture how each of these $L \times A$ potential mutations perturbs the entire $L \times A$ logits matrix. This comprehensive tensor maps every possible mutation to its effect on every possible output score.

To draw residue/nucleotide-level couplings, we first compute the Frobenius norm of the Jacobian $\mathcal{J}$ across the two alphabet-specific dimensions (the letter-dimensions). This reduction yields a two-dimensional interaction map $C \in \mathbb{R}^{L\times L}$:

$$C_{ij} = |\mathcal{J}_{i,:,j,:}|_F^2 \tag{S1}$$

Then, the APC algorithm (Equation 1) is applied to reduce noise.

CJ algorithm is more susceptible to noise than REDIAL for RNAs. While the reduction from the 4-D tensor to 2-D coupling is robust for proteins, where the alphabet size $A = 20$ provides a high-dimensional buffer against local fluctuations, it becomes problematic for RNA ($A = 4$). The $5\times$ smaller vocabulary means that the resulting coevolutionary matrix is more sensitive to noise in the model, leading to weakened signals.

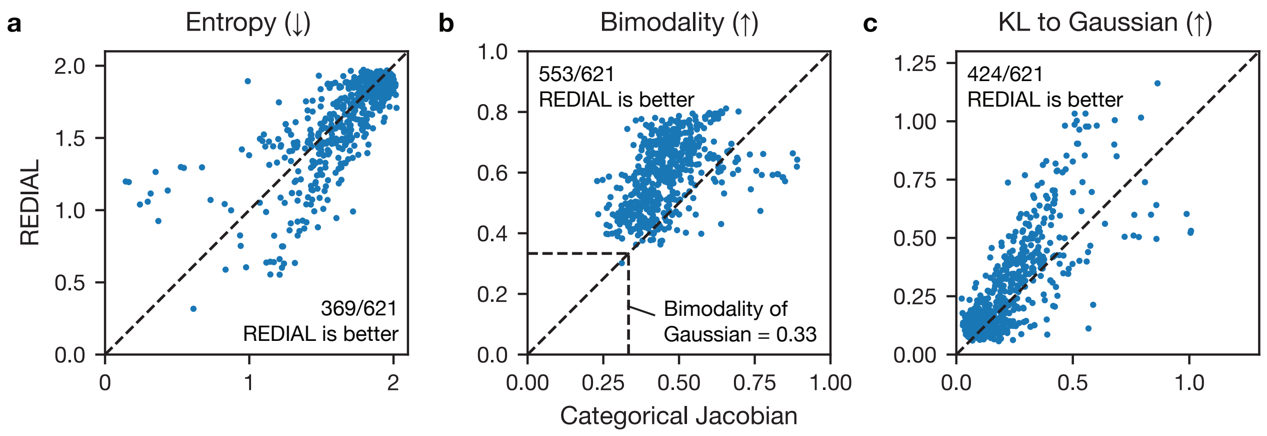

Figure S1. Comparison between Categorical Jacobian method and Embedding Perturbation. Three quantities were computed for the contact maps predicted from NAKB dataset we cleaned. (a) Shannon entropy, (b) Bimodality coefficient, and (c) Kullback-Leibler divergence to a Gaussian.

This higher level of noise can be quantified in several measures. We evaluates how noisy each contact map is by looking at the distributions $p(x)$ of the values in the predicted contact x. First, the predicted couplings were converted to z-score, with zero mean and unity variance. Then, three measures were computed:

bioRxiv preprint doi: https://doi.org/10.64898/2026.05.11.724344; this version posted May 12, 2026. The copyright holder for this preprint (which was not certified by peer review) is the author/funder, who has granted bioRxiv a license to display the preprint in perpetuity. It is made available under a CC-BY 4.0 International license.

Shannon Entropy The Shannon entropy defined as

$$S[p(x)] = -\int p(x) \log_2 p(x) \, dx. \tag{S2}$$

The smaller this value is, the more spread out the distribution is. In an informative contact map, the coupling signals should be distinct from the background. A lower entropy value indicates a more structured, less uniform distribution, reflecting a departure from random noise.

Bimodality coefficient Sarle's Bimodality Coefficient is defined to measure how bimodal a distribution is. The more bimodal, the more informative the predicted contact map may be. It is defined as

$$BC = \frac{\gamma^2 - 1}{\kappa}, \tag{S3}$$

where $\gamma = \langle (x-\mu)^3 \rangle / \sigma^3$ is the skewness (μ for mean, and σ for standard deviation) and $\kappa = \langle (x-\mu)^4 \rangle / \sigma^4$ is the kurtosis. Specifically, a Gaussian distribution has a bimodality coefficient of $1/3$. An ideal contact map should exhibit a bimodal distribution, where a wide "noise" peak is clearly resolved from a "signal" peak corresponding to true physical contacts. A higher bimodality coefficient thus indicates better resolution of structural features.

Kullback-Leibler divergence to a Gaussian This measure how similar this distribution is to a Gaussian $\mathcal{N}(0,1)$, which is different from the Shannon entropy by a cross entropy term. It is defined as

$$H[p(x)] = \int p(x) \ln \frac{p(x)}{\mathcal{N}(0,1)(x)} \, dx. \tag{S4}$$

A larger KL divergence signifies that the predicted map is less likely to be a Gaussian-like stochastic noise, indicating a more robust extraction of non-random evolutionary signals.

While these metrics represent different mathematical properties of the data, in every assessment, the contact maps generated via REDIAL demonstrated statistically better performance compared to the CJ algorithm in RNAs, especially in the bimodality test. This confirms that perturbing the higher-dimensional hidden representation effectively averages out the categorical noise inherent in logit-based extractions more significant in RNA models.

B Results of Direct Coupling Analysis (DCA) for the five systems

The result of the DCA and its multiple sequence alignment (MSA) were summarized in Supplementary Table 2. In the main text, we reported the result from mean field DCA. Here we also show show the result from Pseudolikelihood maximization DCA (Figure S2). Due to the low number of available sequences, they appear much noisier.

Table 2. Statistics on MSA and DCA results.

System	Length	MSA sequences	Effective sequences
1FIR	76	60,573	26.33
1Y26	71	161	5.84
2GDI	80	1,283	10.68
3Q3Z	75	21	5.75
4LVV	85	58	4.88

bioRxiv preprint doi: https://doi.org/10.64898/2026.05.11.724344; this version posted May 12, 2026. The copyright holder for this preprint (which was not certified by peer review) is the author/funder, who has granted bioRxiv a license to display the preprint in perpetuity. It is made available under a CC-BY 4.0 International license.

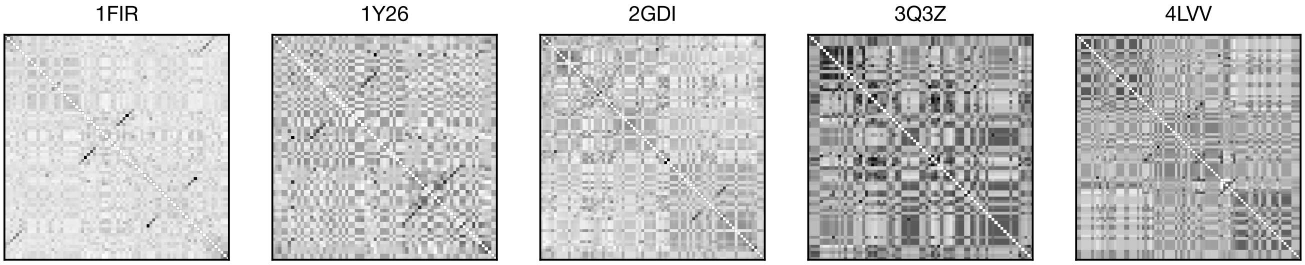

Figure S2. Pseudolikelihood maximization DCA results. Comparing with the meanfield DCA in the main text, these signals are noisier due to small number of available MSA sequences.

C Statistics on Contact PR-AUC scores

The distribution of PR-AUC scores were analyzed in detail here. Figure S3 shows the total distribution shows StructRFM is much better than the vanilla RNA-FM.

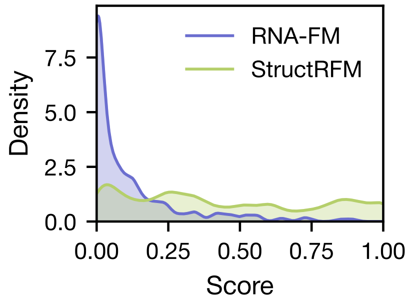

Figure S3. The Contact PR-AUC scores distribution for RNA-FM and StructRFM.

We dissected the scores in four categories and showed their respective performance in Figure S4. In the category of tRNAs, some shorter sequences are from anticodons of tRNAs. They are often much shorter in length.

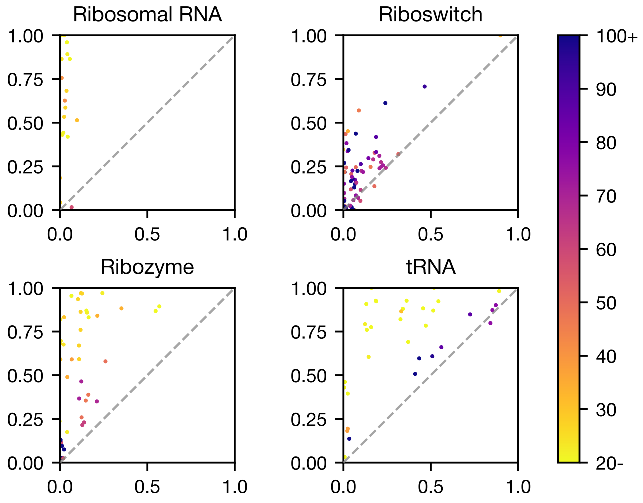

Figure S4. Comparison of contact scores by NAKB annotations. Note that RNAs not in these four categories are not shown here.

bioRxiv preprint doi: https://doi.org/10.64898/2026.05.11.724344; this version posted May 12, 2026. The copyright holder for this preprint (which was not certified by peer review) is the author/funder, who has granted bioRxiv a license to display the preprint in perpetuity. It is made available under a CC-BY 4.0 International license.

We also analyze the distribution of scores by length group in Figure S5. These two models show clear discrepancies in behavior regarding sequence length. StructRFM's performance decreases with length while RNA-FM is consistently not performing.

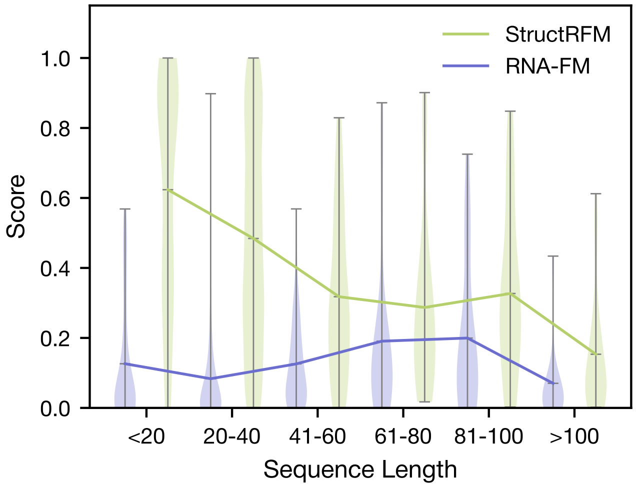

Figure S5. Score distribution violin plot for six length groups StructRFM performs consistently better than RNA-FM. However, the performance of both models struggle with long RNAs with length > 100. StructRFM has the largest margin for short RNAs with length < 20.

Additional Supplementary Figures

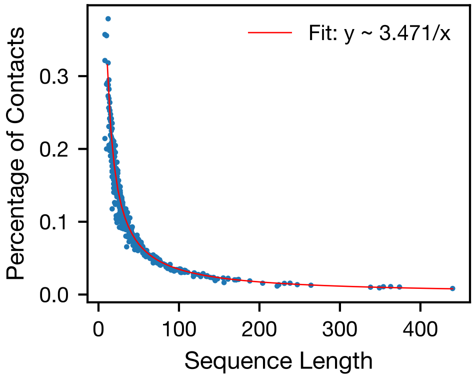

Figure S6. Percentage of contacts vs. sequence length. The percentage of base pairs within 6.5 Å of each other among all pairs. The fit was done with a linear fit between $1/y$ and x. Pearson coefficient $r = 0.9982$.

bioRxiv preprint doi: https://doi.org/10.64898/2026.05.11.724344; this version posted May 12, 2026. The copyright holder for this preprint (which was not certified by peer review) is the author/funder, who has granted bioRxiv a license to display the preprint in perpetuity. It is made available under a CC-BY 4.0 International license.

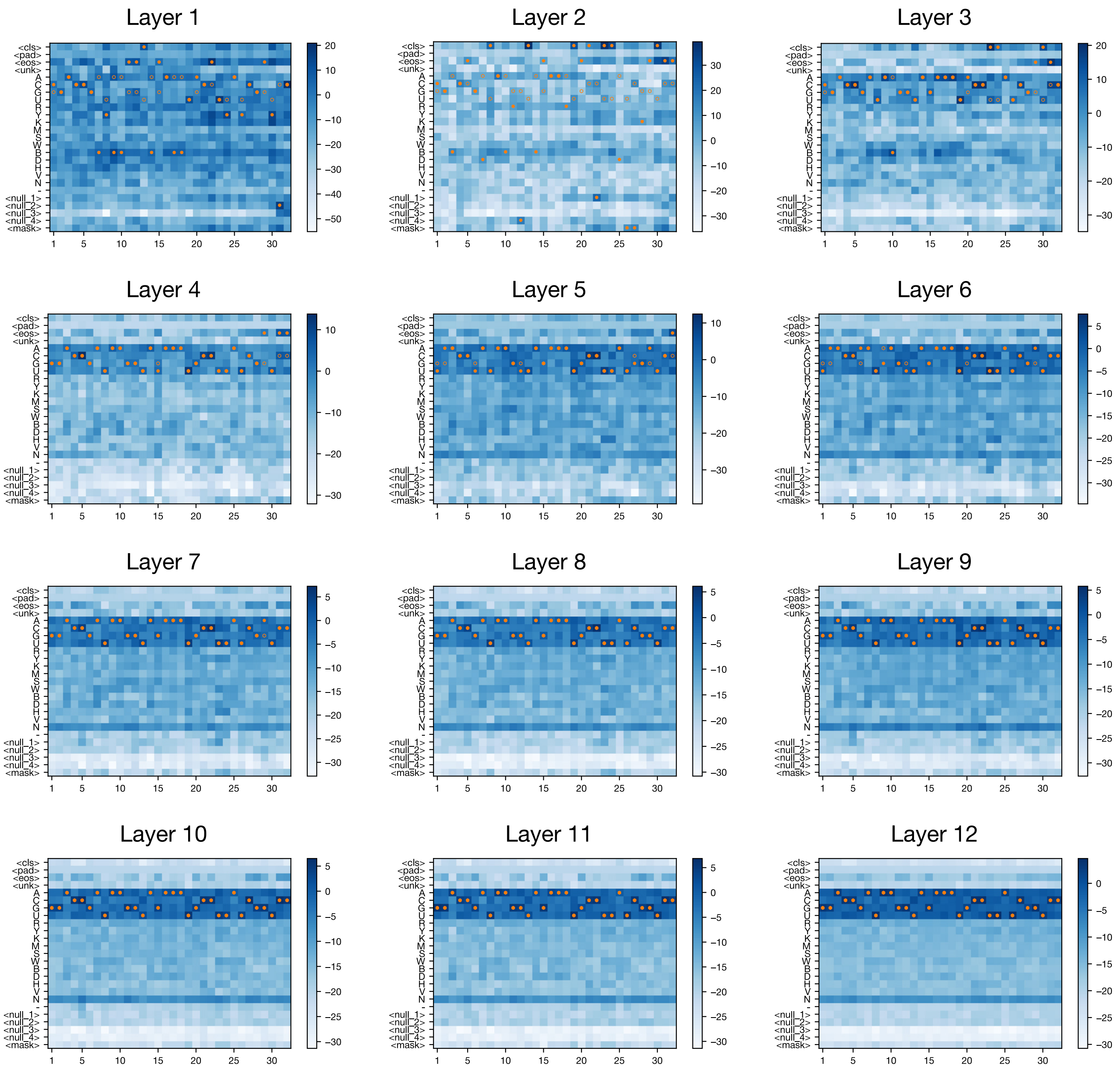

Figure S7. Layerwise decoded logits from `2LI4` and RNA-FM. Hollow point: ground truth sequence. Solid point: predicted token (with largest log-likelihood)
